# Profile of angle closure in a tertiary care center in north India

**DOI:** 10.4103/0301-4738.62643

**Published:** 2010

**Authors:** Parul Ichhpujani, Surinder S Pandav, Aparna Ramasubramanian, Sushmita Kaushik

**Affiliations:** Department of Ophthalmology, Postgraduate Institute of Medical Education and Research, Chandigarh, India

**Keywords:** Angle closure, blindness, epidemiology, glaucoma, North India, primary angle closure, primary angle closure suspect, primary angle closure glaucoma

## Abstract

**Purpose:**

To study the demographic and clinical profile of the types of primary angle closure patients presenting at a tertiary care center in North India.

**Materials and Methods:**

Clinic records of patients diagnosed as primary angle closure were reviewed. International Society of Geographical and Epidemiological Ophthalmology (ISGEO) classification scheme was used to categorize patients. Demographic and clinical data including prior management was collected and analyzed. Main Outcome measures were age, sex, symptomatology, best corrected visual acuity (BCVA), intraocular pressure (IOP), gonioscopy, optic disc assessment and visual field defects. Logistic regression model and receiver operating curve (ROC) were calculated for predictors of type of glaucoma.

**Results:**

Eight hundred and fourteen patients (1603 eyes; males: 380, females: 434) were diagnosed to have various subtypes of angle closure. Mean (±SD) age at presentation was significantly higher for males (57.57 ± 11.62 years) as compared to females (53.64 ± 10.67 years) (*P* < 0001). Primary angle closure glaucoma (PACG) was most frequently diagnosed subtype (49.38%) followed by Primary angle closure (PAC) (39.68%) and Primary angle closure suspect (PACS) (10.93%) respectively. The three subtypes differed significantly among their mean IOP (on ANOVA, F = 14.04; *P* < 0001 using Greenhouse-Geisser correction). Univariate analysis was done to find significant predictors for the outcome of PACG. Logistic regression model and ROC containing the significant predictors yielded a very high AUC of 0.93 with strong discriminatory ability for PACG.

**Conclusion:**

In our hospital-based study, the significant predictors for the outcome of PACG included male gender, diminution of vision, the presence of pain and worsening grades of BCVA. Nearly half of PACG presented with advanced disease. In spite of one-third of the patients being diagnosed as angle closure prior to referral, only 8.34% had iridotomy (laser or surgical) done.

Glaucoma is the second most common cause of visual morbidity after cataract. Worldwide, 60.5 million people are likely to have primary glaucoma by the year 2010; with 8.4 million suffering from bilateral blindness.[1] Asians represent 47% of those with glaucoma and 87% of those with angle closure glaucoma (ACD). Primary angle closure glaucoma (PACG) has been reported to be more prevalent in South East Asian countries than the rest of the world.[2] This was a retrospective study of the angle closure patients attending our glaucoma clinic to analyze the various demographic and clinical aspects of all forms of angle closure. Our center serves as a tertiary care center which caters to states of Punjab, Haryana and Himachal Pradesh; hence it partly reflects the angle closure profile of North India.

## Materials and Methods

A six-and-a-half year retrospective analysis (January 2000-June 2006) of the records of 4703 patients attending glaucoma services at our center was done. Patients found to have angle closure were classified using International Society of Geographical and Epidemiological Ophthalmology (ISGEO) Classification.[2] Approval was obtained from Institutional review board for retrospective data review, analysis and publication. We have reclassified the patients prior to 2002 according to ISGEO Classification by referring to the clinical records. Clinical records were reviewed in detail with respect to presenting complaints, best corrected visual acuity (BCVA), intra ocular pressure (IOP) (by Goldmann applanation tonometer), gonioscopy (using Zeiss 4-mirror goniolens), optic nerve head evaluation and Humphrey threshold 24-2 visual field analysis using Swedish interactive thresholding algorithm (SITA) strategy (Humphrey Instruments Inc., San Leandro, CA). Grading used for gonioscopy was based on structures actually visualized.

Patients with incomplete records and secondary angle closure, such as lens-induced glaucoma, neovascular glaucoma, or uveitis, were specifically excluded.

### Patients were classified into:

*Primary angle closure suspects* (PACS) if an appositional contact was present between the peripheral iris and posterior trabecular meshwork and more than 270 degree of posterior trabecular meshwork could not be visualized.[2]

*Primary angle closure* (PAC) patients had an eye with occludable drainage angle i.e., the posterior (usually pigmented) trabecular meshwork is seen for less than 90° of angle circumference and features indicating that trabecular obstruction by peripheral iris has occurred, such as peripheral anterior synechiae, elevated IOP, iris whorling, “glaucomflecken” lens opacities or excessive pigment deposition on the trabecular surface, with no optic nerve head changes.

*Primary angle closure glaucoma* (PACG) was labeled if disc and field changes were present with PAC (appositional or synechial) as defined above i.e., a vertical cup to disc ratio (VCDR) of 0.7 or greater or asymmetry between the right and left VCDRs of 0.2 or more, and a visual field defect consistent with glaucoma.[2] If the media opacities obscured optic disc assessment, then an IOP greater than 26 mm Hg and visual acuity worse than 20/400, or evidence of previous glaucoma filtering surgery was considered. The VCDR and IOP criteria described above were based on the 97.5^th^ and 99.5^th^ percentiles for “hypernormals” in surveys described by Foster *et al*.[2]

Minimal criteria for labeling a glaucomatous visual field defect were as follows: Glaucoma hemifield test (GHT) outside normal limits, pattern standard deviation (PSD) with *P* values <5%, or a cluster of three or more points in the pattern deviation plot in a single hemifield with *P* values <5%, one of which must have a *P* value <1%. Any one of the preceding criteria, if found again on repeat testing on two tests within one month, was considered sufficient evidence of a glaucomatous visual field defect.[3,4] Visual fields were done for patients with BCVA of 20/200 or better. Advanced field defects were defined as mean deviation greater than -12 dB and on Pattern deviation plot, points below 5% between 37 to 55 with points below 1% ranging from 19 to 36. The diagnosis was confirmed by either of the two senior consultants in all cases. We present our data using both the visual acuity and visual field criteria [World Health Organization (WHO) criteria].[5] Eyes with advanced field damage and/or VCDR of more than 0.80 and/or BCVA less than 20/400 due to glaucomatous disc damage were classified as advanced glaucoma.[6]

Statistical analysis was performed using SPSS statistical software (10.0 version). Data were descriptively analyzed for estimates of quantitative variables using mean and 95% confidence intervals and qualitative variables as proportions. Chi-square test of independence was used to evaluate associations between qualitative variables and Glaucoma Classification groups. Univariate Odds Ratios (with their 95% CI) were calculated for various dichotomous characteristics. ANOVA was used to compare intraocular pressures measured in the eyes affected by three different types of glaucoma (814 patients providing data for 1603 eyes).

Logistic regression analysis was carried out on predictors for PACG found significant on Univariate analysis. All tests were two-tailed and *P*-values <0.05 were taken as significant.

## Results

Of the 4703 patients seen during the specified span, 1505 (32%) presented with primary glaucoma (both structural and functional damage) and ocular hypertension (OHT). POAG was seen in 595 (39.5%) patients, OHT in 96 (6.4%) while 814 (54.1%) patients had PAC subtypes. Since PACS and PAC do not have structural abnormality, the net proportion of PACG was 40.3%.

**Figure 1:**
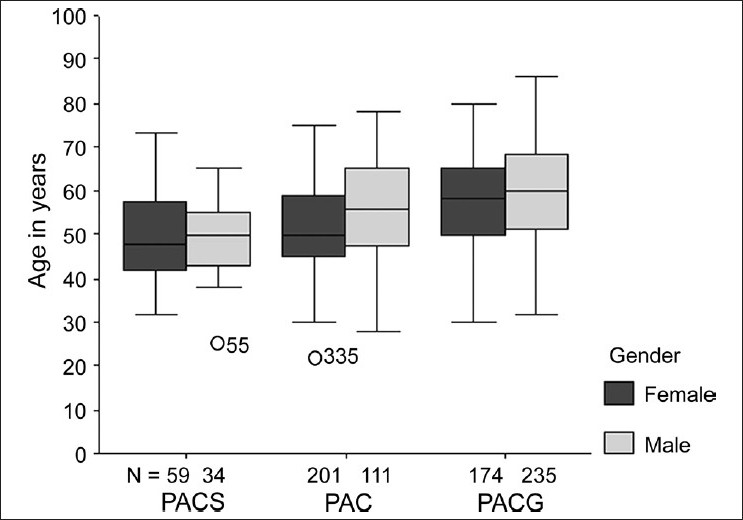
Subtypes of angle closure stratified by gender

Amongst the angle closure subtypes, PACG was the most common seen in 402 patients (n = 814; 49.4%) followed by PAC in 323 patients (39.7%) and PACS in 93 patients (11%). In patients with different subtypes of angle closure in both eyes, the eye with the higher degree of angle closure was used for categorization. PACG had an overall peak presentation in the seventh decade (34.8%) while PAC was more common in the sixth decade (34.1%). The overall mean age at presentation for males was 57.70 years (95% CI; 56.53-58.86); significantly higher when compared to mean age of 53.56 years (95% CI; 52.55-54.57) in females (*P* < 0.001). The mean age of presentation also varied significantly among patients stratified by types of glaucoma [Table 1]. However, on general linear model analysis using age as dependent variable and gender as well as type of glaucoma as fixed covariates, there was no significant group interaction between gender versus type of glaucoma (F = 1.55; *P* = 0.21) as far as mean age of patients was concerned [Fig. 1].

**Table 1:** Comparative analysis of quantitative variables of age and IOP stratified by Glaucoma subtypes by ANOVA usingPosthoc test

	PACS (n=93)	PAC (n=312)	PACG (n=409)	F-value	*P*-value	Posthoc Tukey
						
	Mean (95%CI)	Mean (95%CI)	Mean (95%CI)			
Age in years	49.69	53.36	58.44	34.25	<.0001	PACG>PAC
	(47.64-51.75)	(52.18-54.54)	(57.34-59.53)			PACG>PAC
						PAC>PACS
Eyes assessed	202	730	671			
IOP	PACS	PAC	PACG	F-value*	*P*-value	Post-hoc**
	15.92(15.47-16.37)	17.24(16.51-17.97)	21.06(19.76-22.36)	33.29	<.0001	PACG>PAC
						PACG>PACS
						PAC>PACS

** Posthoc paired t-test was used with Bonferroni correction for multiple comparison

Gender distribution also showed distinctive association among various types of glaucoma patients. In the PACG category, males (235/409; 57.5%) significantly outnumbered females (174/409; 42.5%) (Chi-square 13.30; *P* < 001). In contrast, proportion of females was significantly higher in PACS (59/93; 63%) as well as PAC (201/312; 64.5%).

**Figure 2:**
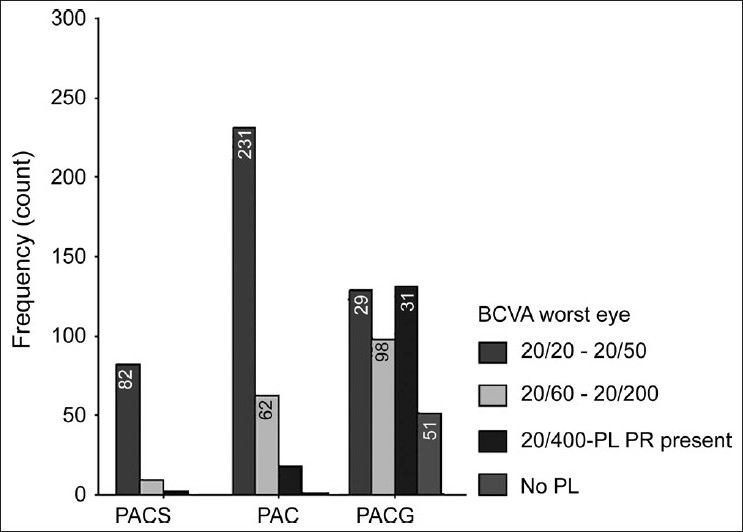
Visual acuity range of subtypes

Diminution of vision was the most common presenting symptom in all the subtypes. Amongst PACS, 88% (82/93) had BCVA between 20/20 to 20/50 while latter was observed only in 74% (231/312) of PAC and 31.5% (129/409) of PACG patients. Fifty two (12.5%) the PACG patients (28 females, 24 males) had no perception of light. Visual acuity between 20/400 to perception of light present was seen in 2.2% (2/93) PACS, 5.8% (18/312) PAC and 32% (131/409) PACG patients [Fig. 2]. Overall, association between these two ordinal measures i.e. glaucoma severity and BCVA grades was found to be highly significant by Kendall's tau-b test (*P* < 0.001). According to the WHO definition of blindness,[5] 46 (5.7%) patients were bilaterally blind and 167 (20.5%) were unilaterally blind. BCVA better than 20/400 was seen in 1335 eyes (84.5%).Though patients with PACS, PAC and PACG differed among their mean IOP (on repeated measure ANOVA, F = 27.37; *P* < 0.001 using Greenhouse-Geisser correction), prior medication (*P* = 0.78) and surgery (*P* = 0.88) had no significant interaction with diagnostic categories of glaucoma as far as their mean IOP values are concerned. The blindness was attributed to advanced glaucomatous optic neuropathy.

In addition, one female patient had central retinal artery occlusion (CRAO) and five had central retinal vein occlusion (CRVO). All had a history suggestive of acute angle closure attack in the past in the affected eye followed by a sudden diminution of vision. Mean IOP at presentation was 18.71 ± 8.43 mmHg in eyes that had received prior medical therapy [228 patients (35.7%)] and 18.47 ± 7.11 mmHg in remaining eyes. Surgical iridectomy or filtering surgery was seen in 33 patients (4.1%) while laser iridotomy (LPI) was noted in 35 patients (4.3%).

Three hundred and fifty six eyes (54.1%) amongst PACG presented with advanced glaucomatous field damage and advanced glaucomatous optic neuropathy (VCDR > 0.8) in 317 eyes (48.2%). Out of 814 angle closure patients, 60 (7.4%) were lost to follow-up and hence visual field analysis could not be performed; baseline fields were unreliable for 79 (9.7%) patients.

**Figure 3:**
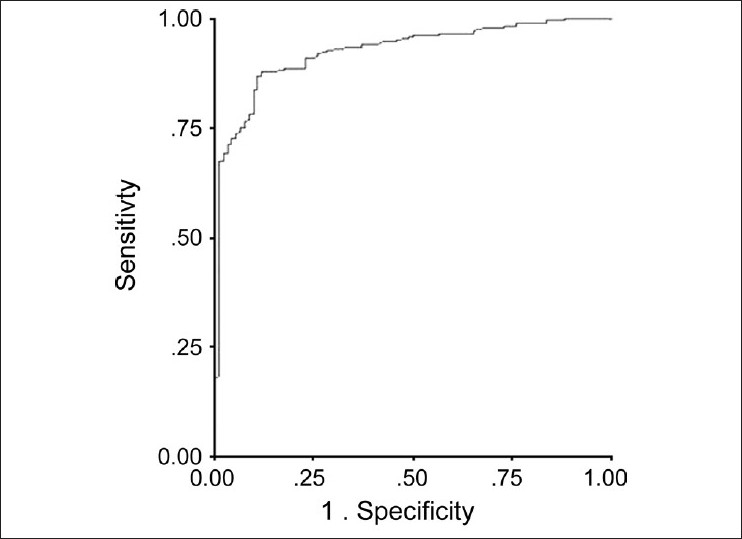
Receiver operating curve showing area under curve (0.93) for outcome of primary angle closure glaucoma

On univariate analysis, the significant predictors (explan atory variables) for the outcome of PACG included male gender, diminution of vision, presence of pain and worsening grades of BCVA (above 20/50) [Table 2]. Patients with diminution of vision had over 15 times the odds of developing PACG as compared to patients with PACS (*P* < 0.001; OR = 15.21; 95%CI: 8.35-27.71). Similarly, diminution of vision increased over nine times the odds of PACG over PAC group (*P* < 0.001; OR: 9.64; 95%CI: 5.86-15.85). The presence of pain also was a predictor of severity of glaucoma; odds higher for PACG in patients with pain as compared to PACS (3.58; 95% CI: 2.23-5.77) as well as PAC patients (2.54; 95% CI: 1.88- 3.45) respectively. On the contrary, odds of colored haloes favored PACS (OR 3.81; 95% CI = 1.90-7.63; *P* < 0.001) as well as PAC (OR 2.93; 95% CI = 1.70-5.05, *P* < 0.001) over the odds of PACG in our study. When subjected further to multivariate logistic regression (predicting presence of PACG and using PACS as the reference category) with backward Logistic Regression method, the above mentioned variables continued to be highly significant predictors of PACG in the final model. Furthermore, receiver operating curve (ROC) of model containing these significant predictors yielded a very high area under curve (AUC) of 0.93 with strong discriminatory ability for PACG [Fig. 3].

**Table 2:** Logistic regression analyses showing patient characteristics as predictors of glaucoma severity (outcome of PACG compared with PACS)

Risk factor	Univariate	95%CI	*P*-value	Multivariate	95%CI	*P*-value
	odds ratios			odds ratios#		
Male	2.34	1.47-3.73	<.0001	1.93	1.04-3.61	0.03
Diminution of vision	15.21	8.35-27.71	<.0001	18.05	7.13-45.68	<.0001
Presence of Pain	3.58	2.23-5.77	<.0001	8.77	3.91-19.65	<.0001
BCVA(ref. group; <20/50)						
<20/200	6.92	3.31-14.45	<.0001	3.56	1.52-8.30	0.003
PLPR+	41.63	10.02-172.87	<.0001	17.05	3.92-74.08	<.0001
No PL*	91.32	12.47-668	<.0001	64.19	8.57-480.44	<.0001
Age in years**				1.05	1.01-1.08	0.001

# Odds ratios were obtained by means of binary logistic regression analysis using ‘PACS’ as the reference category. OR, odds ratio; CI, confidence interval

* Reference category for this predictor was “PAC” as there was no patient in PACS group;

** Since age is a quantitative predictor, only logistic regression odds ratio are mentioned.

## Discussion

The epidemiology of ACG has received marked attention in recent times. Difficulty in estimation of the prevalence of ACG stems from the fact that angle closure is an ongoing process and hence the stage at which patient presents to an ophthalmologist is important. Generally patients characterized by an acute episode or striking symptoms get diagnosed, whereas the more chronic forms tend to go unnoticed.[6] PACS, PAC and PACG are now distinct entities that reflect the severity of the anatomical and functional disorder, according to the ISGEO classification scheme.[2]

Four population-based studies have been conducted in South India[7–10] and one in West Bengal[11] as regards prevalence of glaucoma. The results of these studies provide data about the magnitude of the problem.[10] The aim of this study was to ascertain the prevalence of various subtypes of ACG that present to a tertiary care referral centre in North India. Our study being a retrospective analysis has limitations associated with case record review with added issues of selection bias and referral bias, institute being a tertiary care center.

Hospital-based data from North India reports almost equal prevalence of both primary open angle glalucoma and PACG.[12] Sihota *et al*. studied the clinical profile of the subtypes of primary ACG: Acute, subacute, and chronic, in a prospective study of 500 patients. ACG constituted 45.9% of all primary adult glaucoma seen. In our study, 814 patients (1603eyes) were found to have PAC. ACG constituted 40.3% of all primary adult glaucoma. We also found a marginal female predominance (53.3%) for angle closure, like Sihota *et al*. (51.4%).[12] Chennai Glaucoma Study (CGS) also reported PAC and PACG to be more common in women.[10]

Sihota *et al*. reported ACG occurred maximally in the sixth decade.[12] In our study, 269 (33.04%) patients were less than 50 years old, of which 54 were less than 40 years of age, which is in agreement with the study by Sihota *et al*.

We found PACG to be the most common type seen in 402 patients (49.38%) followed by PAC in 323 patients (39.68%). Amongst other studies, only CGS and West Bengal Glaucoma Study used ISGEO classification. In CGS, the prevalence of PACG, PAC and PACS was 0.9%, 0.71% and 6.3%, respectively. In natural course the likelihood of PACS should be more than PAC and similarly, PAC should be higher than PACG. On the contrary, in a clinic-based study there is always a selection bias, patients come with complaints and most of PACS would be asymptomatic and hence would not seek consultation. So in a clinic based study, a higher PACG prevalence is expected.

In the hospital-based study by Sihota *et al*. 24.8% had acute ACG[12] while at our center, only 31 patients (4.7%) presented with an acute attack. Only four of these patients had evidence of disc damage, rest presented as an acute rise of pressure in PAC patients. We have not taken into account those patients who reported to us after receiving primary anti glaucoma treatment from the referring physician. Hence, this does not project the exact incidence of acute attacks and may account for the discrepancy between the two studies. In addition, there may be differences in the studied populations.

Diminution of vision and pain (periorbital pain or a non-specific headache) were more common in PACG than PAC or PACS. Most patients with PACG presented late in the course of the disease with probable repeated attacks of intermittent angle closure, which would account for pain as the chief presenting complaint. Sihota *et al*. also documented ocular pain to be most common in the acute and subacute groups, 62.1% and 45.5% respectively.[12]

Prior to referral by a general physician or an ophthalmologist, 228 patients (35.96%) had received medical therapy, surgical iridectomy or filtering surgery was done in 33 patients (4.1%) and LPI was noted in 35 patients (4.3%), in our study. This implies that glaucoma was detected in 36% patients (who received anti glaucoma medication); however, LPI or surgical iridotomy was done in only 8.3% of the referred patients. Pilocarpine was prescribed either alone or in combination to 102 patients (44.7%, n = 228) and was discontinued later without doing LPI. Low rate of LPI, either due to nonavailability of the Nd YAG laser or inability to recognize the condition in PAC and/or PACG patients is a matter of concern.

A large proportion of persons presenting to tertiary care centers in North India with angle closure disease have PAC or PACG. Most of the PACG patients present in the advanced stage of the disease. It is important for an ophthalmologist to identify signs of angle closure as, in India, asymptomatic chronic ACG mimicking POAG is common. LPI should be considered for all PAC or PACG patients. A study conducted at Vellore, based on the natural history of PACS reported that as many as 22% may progress to PAC but none progressed to PACG over a span of five years. Accordingly, LPI may not be warranted for PACS per se.[13] Hence, a comprehensive ophthalmic examination holds the key for timely diagnosis of angle closure disease.

To conclude, in a tertiary care center, PACG is the most common presentation amongst various angle closure subtypes with diminution of vision as the most common presenting complaint. Blindness due PACG is common (20.5%, unilateral). Inspite of one-third of patients being diagnosed as angle closure prior to referral, only 8.34% had iridotomy (laser or surgical) done. Hence, there is a need to emphasize the role of laser iridotomy in the management of angle closure glaucoma.
